# ODACH: a one-shot distributed algorithm for Cox model with heterogeneous multi-center data

**DOI:** 10.1038/s41598-022-09069-0

**Published:** 2022-04-22

**Authors:** Chongliang Luo, Rui Duan, Adam C. Naj, Henry R. Kranzler, Jiang Bian, Yong Chen

**Affiliations:** 1 Division of Public Health Sciences, Washington University School of Medicine in St. Louis, St. Louis, MO USA; 2 Department of Biostatistics, Epidemiology and Informatics, Perelman School of Medicine, University of Pennsylvania, Philadelphia, PA 19104 USA; 3 Department of Biostatistics, Harvard T.H. Chan School of Public Health, Boston, MA USA; 4 Department of Pathology and Laboratory Medicine, Perelman School of Medicine, University of Pennsylvania, Philadelphia, PA USA; 5 Department of Psychiatry, Perelman School of Medicine, University of Pennsylvania and the VISN 4 MIRECC, Crescenz VAMC, Philadelphia, PA USA; 6 Department of Health Outcomes and Biomedical Informatics, College of Medicine, University of Florida, Gainesville, FL USA

**Keywords:** Computational biology and bioinformatics, Risk factors

## Abstract

We developed a One-shot Distributed Algorithm for Cox proportional-hazards model to analyze Heterogeneous multi-center time-to-event data (ODACH) circumventing the need for sharing patient-level information across sites. This algorithm implements a surrogate likelihood function to approximate the Cox log-partial likelihood function that is stratified by site using patient-level data from a lead site and aggregated information from other sites, allowing the baseline hazard functions and the distribution of covariates to vary across sites. Simulation studies and application to a real-world opioid use disorder study showed that ODACH provides estimates close to the pooled estimator, which analyzes patient-level data directly from all sites via a stratified Cox model. Compared to the estimator from meta-analysis, the inverse variance-weighted average of the site-specific estimates, ODACH estimator demonstrates less susceptibility to bias, especially when the event is rare. ODACH is thus a valuable privacy-preserving and communication-efficient method for analyzing multi-center time-to-event data.

## Introduction

Real-world data (RWD) such as electronic health records (EHRs) and medical claims, are used increasingly to provide evidence-based support for healthcare decision making^1–3^. The past decade has seen an increasing number of clinical research networks that accumulate and promote the use of large collections of RWD for clinical research. For example, the international Observational Health Data Sciences and Informatics (OHDSI) collaborative^4^, and the national Patient-Centered Clinical Research Network (PCORnet) in the United States^5^, both cover hundreds of millions of patients. These large data consortia provide opportunities to integrate RWD from various healthcare organizations. Multicenter analyses using RWD from these clinical research networks have expanded rapidly because of improved generalizability from more representative population samples and increased statistical power to detect modest associations between exposures and outcomes.

Despite the benefits of multicenter analyses, two major challenges exist for multi-site data integration. First, the direct sharing of patient-level data across institutions may be prohibited, as individual patient-level data are protected by privacy regulations such as the Health Insurance Portability and Accountability Act (HIPAA) in the United States or the European Union’s General Data Protection Regulation (GDPR). Hence many research networks such as OHDSI and PCORnet have adopted a federated model in which patient-level data are stored at local institutions and often only aggregated information are shared across sites^5–7^. Second, data from different sites are often heterogeneous with respect to patient characteristics, data quality, and other unobserved site-specific features. Assuming that a common statistical model is appropriate across all sites may result in biased estimation and poor prediction.

Within these clinical research networks, the abundance of EHRs containing data on patients at multiple time points is especially useful for survival analyses, which model the time to a specific outcome or event of interest. To conduct multicenter survival analyses without sharing patient-level data, a common and convenient approach is meta-analysis, where a weighted average of the local estimates from each site is used. However, when the outcomes or exposures are rare, or the samples at some sites are small, the accuracy of the meta-analysis may be low^8,9^. To obtain more accurate results under these conditions, distributed algorithms have been developed, such as the WebDISCO (a web service for distributed Cox model learning)^10^. Despite providing identical results to that from pooling individual-level data (“lossless”), this algorithm is communication intensive due to its iterative nature, which requires multiple rounds of communications across sites. To balance communication efficiency and estimation accuracy, Shu et al.^11^ proposed a lossless one-shot algorithm for a stratified Cox model that can include only one binary covariate in the model. Huang and Huo^12^ proposed a distributed one-step estimator to improve the accuracy of meta-analysis estimator. Wang et al.^13^ proposed a “divide-and-conquer approach,” aiming to reduce the computational burden when the sample size is extremely large. Duan et al.^9^ proposed a One-shot Distributed Algorithm for Cox model (ODAC) based on the surrogate likelihood approach that relies on patient-level data from a single site and aggregated data from other sites. This algorithm requires aggregated data from only two iterations but obtains estimates close to those resulting from the inclusion of patient-level data from all sites.

Most of these approaches are based on the Cox proportional-hazards model, with a few accounting for between-site heterogeneity. Specifically, in multicenter survival analyses, baseline hazard functions and the distribution of covariates are likely to differ across sites as patients often come from different sub-populations varying in racial/ethnic compositions across geographic regions. Ignoring the heterogeneity across sites could lead to biased estimated associations. Here we propose a distributed algorithm that accounts for site-level heterogeneities in covariate distributions and baseline hazard functions, the One-shot Distributed Algorithm for Cox model with Heterogeneity (ODACH). Compared to the previously described ODAC, which assumes a common baseline hazard function across sites, ODACH assumes heterogeneous baseline hazard functions, and is therefore more flexible and practical in real-world settings. Moreover, unlike ODAC, the use of a constructed surrogate likelihood means that ODACH does not require an extra round of communication regarding the risk set in each site, improving communication efficiency. We illustrate in a simulation study and in a real-world multicenter opioid use disorder study that our proposed algorithm is both a ‘one-shot’ approach and highly accurate (i.e., demonstrates less bias).

## Results

### A one-shot distributed algorithm for cox model with heterogeneity

**Figure 1:**
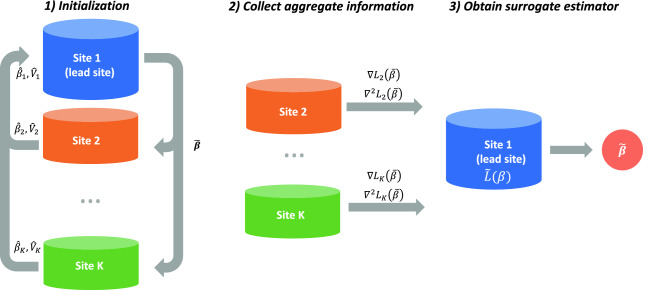
Schematic illustration of the ODACH algorithm. The first step is initialization, where each site reports the local estimation of the log hazard ratio (${\widehat{\beta }}_{j}$) and the corresponding variance estimate (${\widehat{V}}_{j}$). The lead site then computes the initial estimate $\overline{\beta }$ as the weighted average of all local estimates and sends it back to each site. In the second step, each site calculates and shares the local gradients $\nabla {L}_{j}(\overline{\beta })$ and ${\nabla }^{2}{L}_{j}(\overline{\beta })$. In the third step, the lead site constructs a surrogate likelihood function $\tilde{L }(\beta )$ with these gradients and obtains the surrogate estimate $\stackrel{\sim }{\beta }$.

The proposed ODACH algorithm constructs a surrogate log-likelihood function to approximate the log-likelihood function of the stratified Cox model, which is commonly used to account for site-specific baseline hazards when analyzing multi-site time-to-event outcomes. We provide a schematic illustration of the ODACH algorithm in Fig. 1.

### ODACH can reduce estimation bias in multicenter survival analyses

We used a simulation study to demonstrate the bias-reduction property of the proposed ODACH algorithm in multicenter survival analyses, especially when the outcome is rare***.*** We generated time-to-event outcomes that are associated with two covariates. The pooled data are evenly distributed to *K* = 10 clinical sites. Details of the data generation are in the “Methods” section. We applied three approaches to estimate the HRs of the two covariates on the time-to-event outcome, i.e., pooled stratified Cox regression, meta-analysis, and the proposed ODACH method. Because the pooled Cox regression estimator can be considered a gold standard, the relative bias of meta-analysis and ODACH estimates to the pooled estimate are compared to demonstrate the advantage of ODACH.

**Figure 2:**
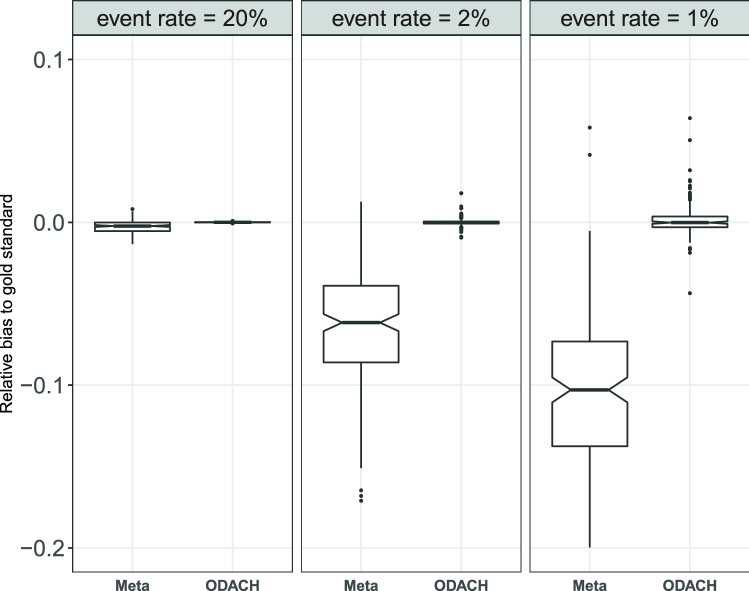
Boxplot of bias relative to the gold standard (stratified Cox model on the pooled dataset across all sites). The two methods compared in the plot are meta-analysis (meta) and One-shot Distributed Algorithm for Cox model with Heterogeneous baseline hazards (ODACH). The event rate varies from 20 to 1% and under each setting the boxplots are based on 200 replications of the simulation. The true effect size is 1.

Results of the simulation show that ODACH achieves better estimation performance than the meta-analysis estimator, especially for a rare event. Figure 2 shows that ODACH yields relative biases close to 0, meaning that it provides results almost identical to those of the pooled estimator, i.e., by stratified Cox model on the pooled dataset across all sites. As the event becomes rarer, the meta-analysis estimator is observed to have a larger bias. For example, when the event rate is 1%, the average relative bias is around − 11% for the meta-analysis estimator, but is negligible for the ODACH estimator. Moreover, the variation of the meta-analysis estimator is much larger than that of the ODACH estimator.

### The OneFlorida opioid use disorder study

We demonstrate the use and advantage of the proposed ODACH method by studying the association of time to an opioid use disorder (OUD) diagnosis with risk factors (e.g., patients’ demographic and clinical characteristics) using RWD from the OneFlorida Clinical Research Consortium. A detailed description of the data and the risk factors are in the “Methods” section.

**Figure 3:**
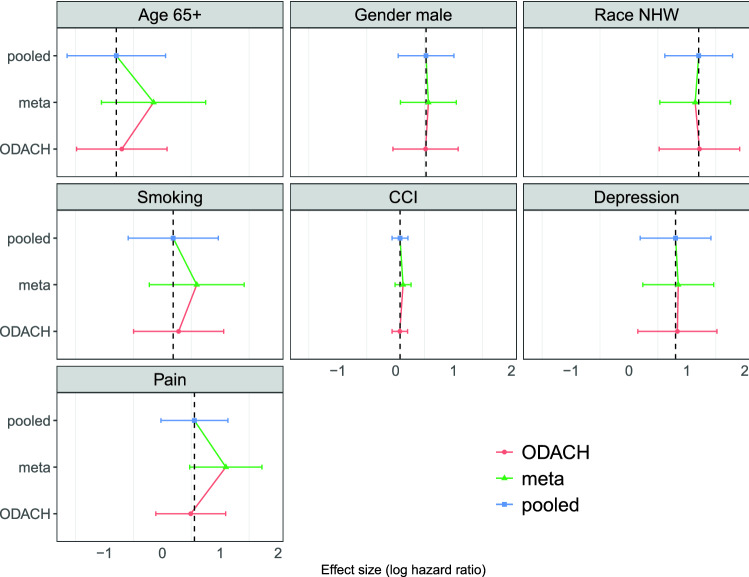
Comparison of estimation biases by meta-analysis and ODACH in the opioid use disorder study. Presented are the estimated log hazard ratios (HRs) with 95% confidence intervals for risk factors for opioid use disorder using pooled analysis (blue), meta-analysis (green), and One-shot Distributed Algorithm for multicenter Cox proportional hazards model with heterogeneous hazard (ODACH) (red). The analyses used data of N = 14,015 patients from five clinical sites in the OneFlorida Clinical Research Consortium.

Figure 3 shows the estimated log HRs of the seven risk factors from the pooled analysis (stratified Cox model), meta-analysis, and the proposed ODACH analysis and their 95% confidence intervals (CIs). The ODACH provides HR estimates that are nearly identical to the pooled estimates for all of the risk factors. As a comparison, meta-analysis estimates have substantial biases relative to the pooled estimator, especially for the effects of age, smoking status, and pain history. For example, the estimated log HR of pain history is 0.554 from the pooled analysis, 1.097 from the meta-analysis, and 0.491 from the ODACH estimator. The relative bias is 98.0% for the meta-analysis estimator and − 11.4% for the ODACH estimator. Moreover, the quantitatively larger biases of meta-analysis estimates may lead to qualitatively different statistical significance. For example, with a significance threshold α = 0.05, the effect of pain history on time to OUD diagnosis is considered statistically significant (p = 0.001) per the meta-analysis estimator, but not statistically significant per either the pooled analysis (p = 0.061) or the ODACH estimator (p = 0.111).

## Discussion

We developed a privacy-preserving One-shot Distributed Algorithm for the Cox model to analyze Heterogeneous multicenter time-to-event data (ODACH). The proposed surrogate likelihood approach approximates the log partial likelihood of the stratified Cox model that uses patient-level data from all of the sites. The simulation study and application to the real-world OneFlorida OUD study both show that the surrogate estimation yields results that are closer than the typical meta-analysis approach to the pooled analysis results, especially when the event is rare. As suggested by a reviewer, simulation results comparing more approaches are deferred in the Supplementary Information. Compared to the existing One-shot Distributed Algorithm for Cox model (ODAC), ODACH allows baseline hazard functions and covariate distributions to be site specific, and hence it is more flexible in its application.

RWD play an increasing role in generating real-world evidence to support healthcare decision making. Observational RWD such as those from EHRs and medical claims contain longitudinal information, which enables time-to-event modeling such as through the Cox proportional-hazards model, one of the most commonly used models for time-to-event analysis in observational studies that evaluate treatment effects and identify risk factors. In multicenter studies, when sharing patient-level data across databases is not possible, the individual estimates from each database are integrated through a meta-analysis approach. Our proposed distributed algorithm could provide a better alternative to the commonly used meta-analysis, with particular benefits in the case of rare events. The algorithm is implemented in the R package “*pda*”^14^. A demo example is available at https://github.com/Penncil/ODACH.

There are several directions for future work. For instance, time-varying covariates or time-varying effects are sometimes encountered in time-to-event analyses^15,16^. Under these conditions, because the Cox model relaxes the usual proportional hazards assumption but requires additional data for accurate estimation^17,18^, the development of a distributed algorithm for the Cox model with time-varying covariates or time-varying effects in multi-center studies would be desirable. Moreover, in certain settings, other survival models such as the accelerated failure time (AFT) model^19^ are more appropriate than the Cox model. A distributed algorithm for the AFT model is currently under investigation and will be reported in the future. In addition, because sources of heterogeneity other than baseline hazard functions or distributions might exist, such as missing data patterns and site-specific effect sizes, robust methods for handling different types of heterogeneity^20–22^ are needed to avoid potentially misleading results.

## Methods

### The ODACH algorithm

Suppose that we have study subjects from $K$ different clinical sites and denote ${n}_{j}$ to be the number of subjects in the *j*-th site. We denote the total number of subjects as $N=\sum_{j=1}^{K}{n}_{j}$. For the *i*-th subject in the *j*-th site, we observe $\{{T}_{ij}, {\delta }_{ij}, {x}_{ij}\}$, where ${T}_{ij}$ is the observed time to event, ${x}_{ij}$ is a p-dimensional covariate variable, and ${\delta }_{ij}=0$ indicates censoring and ${\delta }_{ij}=1$ indicates an event. The Cox proportional hazard model describes that the hazard of the *i*-th subject in the *j*-th site having the event at time $t$ as $\lambda \left(t|{x}_{ij}\right)={\lambda }_{j}\left(t\right)\mathrm{exp}\left({\beta }^{T}{x}_{ij}\right)$. We assume that the log hazard ratio (HR) $\beta$ is the same across all sites, i.e., there are common effects of the covariates on the time-to-event across sites. The stratified log Cox partial likelihood function is

1 $$L\left(\beta \right)= \frac{1}{N}\sum_{j=1}^{K}{{n}_{j}L}_{j}(\beta ),$$

where ${L}_{j}\left(\beta \right)$ is the log Cox partial likelihood function for the *j*-th site,

2 $${L}_{j}\left(\beta \right)=\frac{1}{{n}_{j}}\sum_{i=1}^{{n}_{j}}{\delta }_{\mathit{ij}}\mathrm{log}\frac{\mathrm{exp}\left({\beta }^{T}{x}_{ij}\right)}{{\sum }_{s\in {R}_{j}\left({T}_{ij}\right)}\mathrm{exp}\left({\beta }^{T}{x}_{sj}\right)},$$

where ${R}_{j}\left(t\right)$ is the risk set in site *j* at time *t* defined as ${R}_{j}\left(t\right)=\{i;{T}_{ij}\ge t\}$, which contains all of the subjects in site *j* who have not experienced an event or been censored at time $t$. The common effect $\beta$ can be estimated by maximizing (1), i.e., $\widehat{\beta }=argma{x}_{\beta } L(\beta )$. We call this the pooled estimator, as it requires all of the data to be pooled together.

In practice, it is often difficult to transfer patient-level data across sites, hence the pooled estimate $\widehat{\beta }$ can be hard to obtain. Inspired by the previously-developed surrogate likelihood approach^8,9,23^, we aimed to construct a proxy of the stratified Cox partial likelihood function (1), using only summary-level information from other sites. We assume we have access only to the patient-level data from a lead site (e.g., the first site). The ODACH surrogate likelihood function is constructed as

3 $$\tilde{L }\left(\beta \right)= {L}_{1}\left(\beta \right)+\langle \nabla L\left(\overline{\beta }\right)-\nabla {L}_{1}\left(\overline{\beta }\right),\beta \rangle +\frac{1}{2}{\left(\beta -\overline{\beta }\right)}^{T}\{{\nabla }^{2}L\left(\overline{\beta }\right)-{\nabla }^{2}{L}_{1}\left(\overline{\beta }\right)\}\left(\beta -\overline{\beta }\right),$$

where ${L}_{1}(\beta )$ is the log-likelihood function of the lead site, and $\nabla$ and ${\nabla }^{2}$ denote the first and second order gradients of a function (explicit forms of $\nabla {L}_{j}\left(\overline{\beta }\right)$, $\nabla L\left(\overline{\beta }\right), {\nabla }^{2}{L}_{j}\left(\overline{\beta }\right)$ and ${\nabla }^{2}L\left(\overline{\beta }\right)$ can be found in the Supplementary Materials). $\overline{\beta }$ is an initial value that is close to the true value of β, e.g. the inverse variance-weighted average of the estimates obtained by fitting a Cox model at each site,

4 $$\overline{\beta }={\left(\sum_{j=1}^{K}{\widehat{V}}_{j}^{-1}\right)}^{-1}\sum_{j=1}^{K}{\widehat{V}}_{j}^{-1}{\widehat{\beta }}_{j},$$

where ${\widehat{\beta }}_{j}= {\mathrm{argmax}}_{\beta }{L}_{j}\left(\beta \right)$ is the estimator of the Cox model fitted on data at the $j$-th site, and ${\widehat{V}}_{j}$ is the estimated variance of ${\widehat{\beta }}_{j}$. The surrogate estimator is thus obtained by maximizing (3), i.e., $\stackrel{\sim }{\beta }=argma{x}_{\beta } \tilde{L }(\beta )$.

Intuitively, the surrogate likelihood function (3) modifies the likelihood function ${L}_{1}\left(\beta \right)$ of the lead site to approximate the stratified likelihood (1), with the modification being the first- and second-order terms, i.e., $\langle \nabla L\left(\overline{\beta }\right)-\nabla {L}_{1}\left(\overline{\beta }\right),\beta \rangle$ and $\frac{1}{2}{\left(\beta -\overline{\beta }\right)}^{T}\{{\nabla }^{2}L\left(\overline{\beta }\right)-{\nabla }^{2}{L}_{1}\left(\overline{\beta }\right)\}\left(\beta -\overline{\beta }\right)$. By sharing the second-order gradients, our method allows each site to have different covariate distributions. In the construction of the surrogate likelihood function (3), ${\nabla }^{\mathrm{r}}L\left(\overline{\beta }\right)$ can be calculated distributively by ${\nabla }^{r}L\left(\overline{\beta }\right)= \frac{1}{N}\sum_{j=1}^{K}{n}_{j}{\nabla }^{r}{L}_{j}\left(\overline{\beta }\right)$, for $r=1, 2$. Because $\nabla {L}_{1}\left(\overline{\beta }\right)$ and ${\nabla }^{2}{L}_{1}\left(\overline{\beta }\right)$ are available from the lead site, it only requires other collaborative sites to calculate and transfer $\nabla {L}_{j}\left(\overline{\beta }\right)$ and ${\nabla }^{2}{L}_{j}\left(\overline{\beta }\right), j=2,\dots , K.$ As these gradients are all aggregated information, patient-level information is protected. We summarize the ODACH algorithm in the box below.

Note that we assume the first site is the lead site when constructing the surrogate likelihood. In practice, if any site can serve as the lead site, we recommend using the largest site for this purpose. Alternatively, after the derivatives ${\nabla }^{\mathrm{r}}{L}_{j}\left(\overline{\beta }\right), r=\mathrm{1,2}, j=\dots ,K$ have been shared across sites, each site can serve as the lead site and obtain its own surrogate estimate. These surrogate estimates can be further synthesized to obtain more accurate estimation.
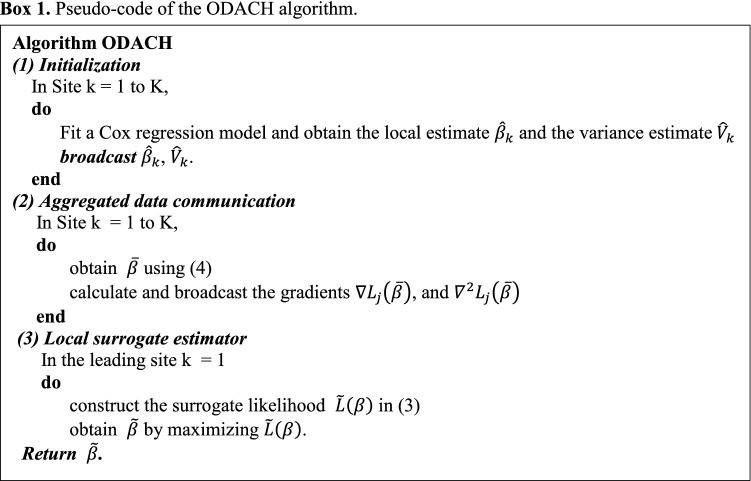


### Simulation study

**Figure 4:**
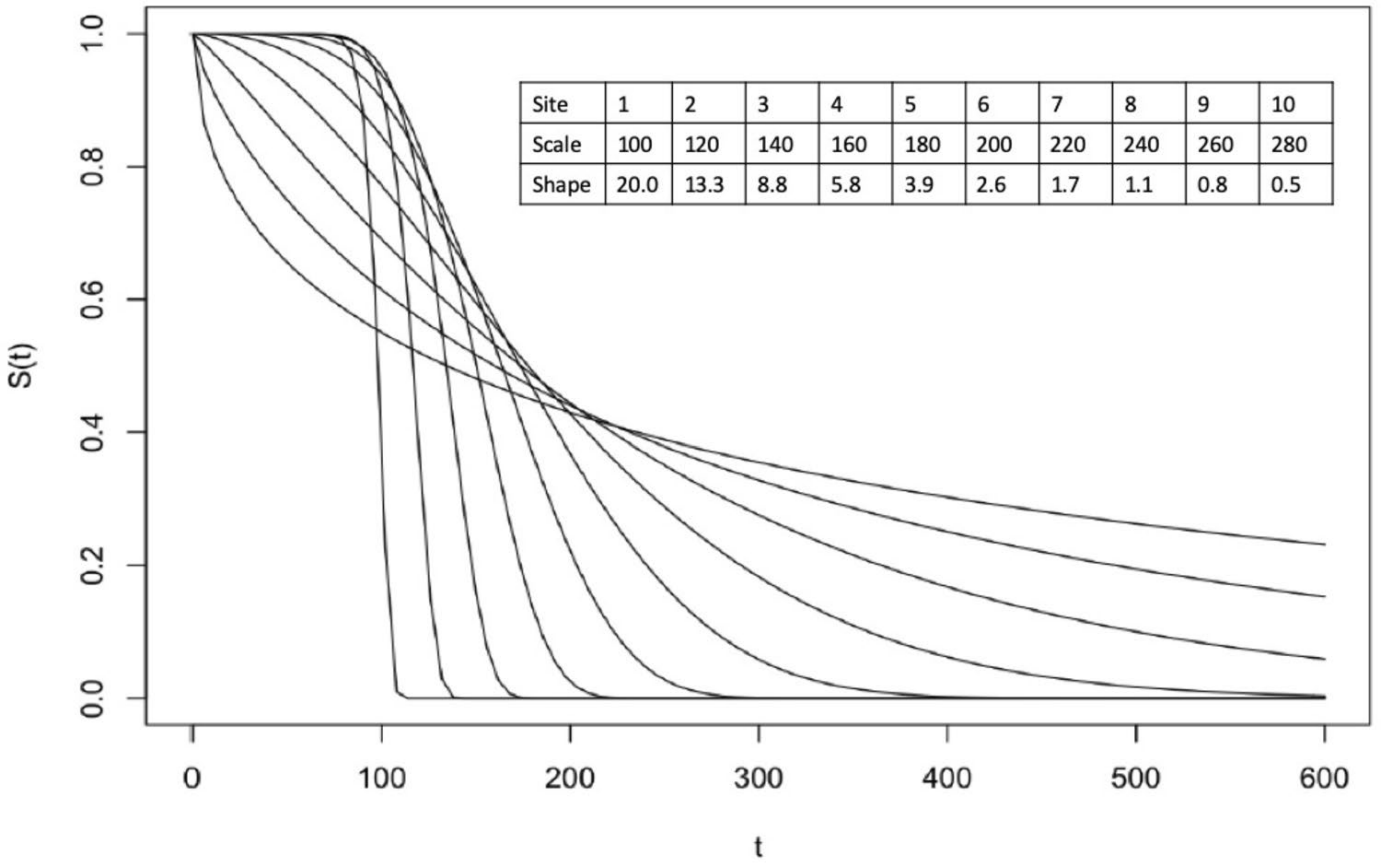
The baseline survival functions of the 10 sites in the simulated data. The varying hazard functions are Weibull functions with scale and shape parameters as listed.

We evaluated the performance of the proposed ODACH estimator using simulated multi-site time-to-event data. A pooled dataset of N = 5000 subjects was generated based on a Weibull proportional hazards model, where the baseline hazard follows a Weibull distribution with varying scale and shape parameters. Specifically, the scale parameters range from 100 to 280 and are equally spaced. The shape parameters range from 20 to 0.5, spaced equally in the logarithmic scale (see Fig. 4 for an illustration). We generated two covariates from uniform distributions and the true log HRs were set to be $\beta$ = (− 1, 1). We set the event rate (number of cases over number of subjects) as 20%, 2% or 1% by generating censoring times following appropriate distributions. The pooled data were evenly distributed to K = 10 clinical sites, with 500 subjects in each site. We applied three approaches to estimate the HRs of the two covariates on the time-to-event outcome, i.e., pooled stratified Cox regression, meta-analysis, and the proposed ODACH method. Because the pooled Cox regression estimator can be considered a gold standard, the relative bias of meta-analysis and ODACH estimates to the pooled estimate are compared to demonstrate the advantage of ODACH. The simulation was replicated 200 times. For simplicity of illustration, we present only the results for the estimation of coefficient ${\beta }_{2}$, as the results for the other coefficient are similar.

### The OneFlorida opioid use disorder study

We evaluated the use and advantage of the proposed ODACH method by studying the association of time to an opioid use disorder (OUD) diagnosis with risk factors using RWD from the OneFlorida Clinical Research Consortium. A total of 14,015 subjects were sampled from five clinical sites and followed for 3 years after their index opioid prescription for chronic non-cancer pain (CNCP) and the time to the diagnosis of OUD was recorded. A summary of the patients’ age (65 + vs. 18–65), gender (male vs. female), race (Non-Hispanic White (NHW) vs. others), smoking status (current smoker vs. others), CCI (Charlson comorbidity index^24^, a weighted score of comorbid conditions), depression, and pain history measured at the index date are shown in Table 1. The rates of OUD are < 1% at all sites.

**Table 1:** Characteristics of the patients from five *OneFlorida* clinical sites.

Site	Site 1	Site 2	Site 3	Site 4	Site 5
Total, N (%)	4078 (100)	3354 (100)	2367 (100)	2296 (100)	1920 (100)
Age ≥ 65 years, N (%)	602 (14.8)	562 (16.8)	464 (19.6)	433 (18.9)	229 (11.9)
Male, N (%)	1560 (38.3)	1142 (34)	972 (41.1)	799 (34.8)	530 (27.6)
NHW, N (%)	2510 (61.5)	1643 (49)	234 (9.9)	1406 (61.2)	889 (46.3)
Current smoker, N (%)	714 (17.5)	61 (1.8)	1 (0)	297 (12.9)	99 (5.2)
CCI, mean (S.D.)	0.86 (1.64)	0.69 (1.39)	0.97 (1.82)	0.79 (1.56)	0.75 (1.35)
Depression, N (%)	415 (10.2)	196 (5.8)	232 (9.8)	262 (11.4)	155 (8.1)
Pain, N (%)	636 (15.6)	392 (11.7)	252 (10.6)	248 (10.8)	385 (20.1)
OUD, N (%)	19 (0.5)	15 (0.4)	11 (0.5)	11 (0.5)	12 (0.6)

*NHW* non-Hispanic White, *CCI* Charlson comorbidity index, *OUD* opioid use disorder.

### Use of experimental animals, and human participants

The use of human subject HIPAA limited data set was approved by the University of Florida (UF) Institute Review Board (IRB) under the protocol number IRB202001100. The University of Florida Federalwide Assurance number is FWA00005790. The study protocol has been reviewed by the UF IRB in accordance with the institutional and federal guidelines. Both Waivers of Informed Consent and HIPAA Waiver of Authorization were granted by the Institutional Review Board of the University of Florida.

## Supplementary Information

Supplementary Information.
