# Supplementary material for: ODACH: a one-shot distributed algorithm for Cox model with heterogeneous multi-center data
Source: Sci Rep. 2022 Apr 22;12:6627. doi: 10.1038/s41598-022-09069-0 (PMC9033863; doi:10.1038/s41598-022-09069-0)
Supplement: Supplementary file 1 — Supplementary Information. [file 41598_2022_9069_MOESM1_ESM.docx]

**Supplementary Materials for “ODACH: A One-shot Distributed Algorithm for Cox model with Heterogeneous Multi-center Data”**

**Details of surrogate likelihood construction**

1. The local first order gradient $\nabla L_{j}\left( \bar{\beta} \right)$

$\nabla L_{j}\left( \bar{\beta} \right)= \frac{1}{n_{j}}\sum_{i=1}^{n_{j}} \delta_{ij}\{x_{ij}-\frac{\sum_{s\in R_{j}(T_{ij})} \exp\left( \bar{\beta}^{T}x_{sj} \right)x_{sj}}{\sum_{s\in R_{j}(T_{ij})} \exp(\bar{\beta}^{T}x_{sj})}\}$ (S.1)

1. The local second order gradient $\nabla^{2}L_{j}\left( \bar{\beta} \right)$

$\nabla^{2}L_{j}\left( \bar{\beta} \right)= \frac{1}{n_{j}}\sum_{i=1}^{n_{j}} \delta_{ij}[ \frac{\left\{ \sum_{s\in R_{j}\left( T_{ij} \right)} \exp\left( \bar{\beta}^{T}x_{sj} \right)x_{sj} \right\}^{\otimes2}}{\left\{ \sum_{s\in R_{j}\left( T_{ij} \right)} \exp\left( \bar{\beta}^{T}x_{sj} \right) \right\}^{2}}-\frac{-\sum_{s\in R_{j}\left( T_{ij} \right)} \exp\left( \bar{\beta}^{T}x_{sj} \right)x_{sj}^{\otimes2}}{\sum_{s\in R_{j}\left( T_{ij} \right)} \exp\left( \bar{\beta}^{T}x_{sj} \right)}]$ (S.2)

where $a^{\otimes2}$ for a vector $a$ denotes the outer product $aa^{T}$.

1. The global first order gradient $\nabla L\left( \bar{\beta} \right)$

$\nabla L\left( \bar{\beta} \right)= \frac{1}{N}\sum_{j=1}^{K} n_{j}\nabla L_{j}\left( \bar{\beta} \right)$ (S.3)

1. The global second order gradient $\nabla^{2}L\left( \bar{\beta} \right)$

$\nabla^{2}L\left( \bar{\beta} \right)= \frac{1}{N}\sum_{j=1}^{K} \nabla^{2}L_{j}\left( \bar{\beta} \right)$ (S.4)

**Comparison with more approaches**

In addition to the simulation study in the main manuscript*,* we include two more approaches in the comparison. The first approach is via the direct approximation of the stratified log Cox partial likelihood function by quadratic expansion (i.e., 2^nd^ order Taylor series expansion) of the collaborative sites, i.e. by optimizing the following surrogate likelihood

$\tilde{L}_{taylor}\left( \beta\right)=\frac{n_{1}}{N}L_{1}\left( \beta\right)+\left\langle\nabla L\left( \bar{\beta} \right)-\frac{n_{1}}{N}\nabla L_{1}\left( \bar{\beta} \right),\beta\right\rangle+\frac{1}{2}\left( \beta-\bar{\beta} \right)^{T}\{\nabla^{2}L\left( \bar{\beta} \right)-{\frac{n_{1}}{N}\nabla}^{2}L_{1}\left( \bar{\beta} \right)\}\left( \beta-\bar{\beta} \right),$ (S.5)

Due to its similarity with the ODACH surrogate likelihood, we name it as ODACH-Taylor approach and the estimate is $\tilde{\beta}_{taylor}$*.* Another approach is via the one-step estimator by Huang and Huo^1^, denoted as

$\tilde{\beta}_{onestep}={\bar{\beta}-\left( \nabla^{2}L\left( \bar{\beta} \right) \right)}^{-1}\nabla L\left( \bar{\beta} \right).$ (S.6)

This estimator does not distinguish the leading site from other collaborative sites, and it is essentially a one-step Newton-Raphson update. We notice that both of the two approaches above aim to improve the initial estimator $\bar{\beta}$ (e.g. a meta-estimator), and both require the same aggregated data from the sites as ODACH (i.e., the same communication cost as the proposed ODAH algorithm).

We then use the same simulation settings to evaluate the relative biases of these approaches (meta, ODACH, ODACH-Taylor and One-step) to the pooled analysis. The results are summarized in Figure S1. The ODACH-Taylor and One-step estimators perform similarly and both improved the meta-estimator towards less bias, and the improvements were substantial when the event rate is lower. On the other hand, when the event is rare (e.g. 1%), the two approaches still have noticeable biases (around 2%), while the ODACH estimator has negligible bias.

**Figure S1.** **Boxplot of bias relative to the gold standard (stratified Cox model on the pooled dataset across all sites).** The methods compared are meta-analysis (Meta), One-shot Distributed Algorithm for Cox model with Heterogeneous baseline hazards (ODACH), Taylor-expansion approximation of the collaborative sites (ODACH-Taylor), and the One-step estimator. The event rate varies from 20% to 1% and under each setting the boxplots are based on 200 replications of the simulation. The true effect size is 1.

The ODACH-Taylor and One-step estimator approaches achieve great bias-reduction compared to the meta-analysis approach. The difference in bias-reduction performance between them with ODACH is due to the different ways of approximating the overall stratified likelihood. Compared with the ODACH surrogate likelihood (S.7, copied from eq (3) in the main manuscript),

$\tilde{L}\left( \beta\right)= L_{1}\left( \beta\right)+\left\langle\nabla L\left( \bar{\beta} \right)-\nabla L_{1}\left( \bar{\beta} \right),\beta\right\rangle+\frac{1}{2}\left( \beta-\bar{\beta} \right)^{T}\{\nabla^{2}L\left( \bar{\beta} \right)-\nabla^{2}L_{1}\left( \bar{\beta} \right)\}\left( \beta-\bar{\beta} \right),$ (S.7)

the ODACH-Taylor surrogate likelihood (S.5) utilizes less of the higher-order information of the leading site $L_{1}\left( \beta\right)$, and the one-step estimator completely ignores it. As a result, these two approaches (ODAH-Taylor and one-step estimator) can still be further improved by ODACH when the event is rare. The practical comparison between ODACH-Taylor, One-step estimator and ODACH may depend on factors such as the event rate, sample size per site, number of sites, among others.

**References**

1. Huang C, Huo X. A distributed one-step estimator. *Math Program*. 2019;174(1):41-76.
